# Association of self-reported consumption of cooked meat, fish, seafood and eggs with prostate cancer risk among Nigerians

**DOI:** 10.1186/1750-9378-4-S1-S6

**Published:** 2009-02-10

**Authors:** Flora A Ukoli, Khandaker Taher, Eruke Egbagbe, Mbeja Lomotey, Temple Oguike, Phillip Akumabor, Usifo Osime, Derrick Beech

**Affiliations:** 1 Department of Surgery, Meharry Medical College, Nashville, TN, 37208, USA; 2 Department of Surgery, University of Benin, Benin-City, Edo State, Nigeria

## Background

The observation that the prevalence of latent PCa at autopsy is similar for African-American and African populations [1], and that Asian populations record latent PCa rates comparable to those of U.S. whites [2], despite large geographical differences in PCa incidence world wide, supports the suggestion that environmental cancer 'promoting' factors play a more important role than cancer 'initiating' factors in the etiology of clinically significant PCa [3,4]. Epidemiological studies have demonstrated that dietary animal fat and high energy intake are associated with increased PCa risk, while dietary marine fat is negatively associated with this risk [5]. Higher meat intake is consistently reported to be associated with increased PCa, possibly due to heterocyclic amines such as 2-amino-1-methyl-6-phenylimidazo [4,5-b]pyridine [PhIP], polycyclic aromatic hydrocarbons such as benzo [a]pyrene [BaP], and alpha-methylacyl-CoA racemase, produced in the process of grilling or frying red meat [6]. High consumption of cooked processed meats has also been reported to contribute to the high burden of PCa risk among African-Americans [7]. In China, a low-incidence region for PCa, the consumption of salted fish and preserved meats has been reported to be associated with a significant increase in PCa risk [8]. Current evidence from cohort studies supporting the association between high fish intake with reduced PCa risk is however less convincing for countries with low or high fish consumption [9,10]. Meat, fish, cheese and egg intake were not associated with PCa risk in a Netherland cohort study [11]. Like other Sub-Saharan designated low-incidence regions for PCa, Nigeria has reported an moderate upward incidence trend, with PCa becoming the most diagnosed male cancer [12,13]. This trend is postulated to result from improved diagnosis, increased longevity, and the progressive replacement of their traditional low-fat diet with a more westernized diet high in meat and processed foods. This study examined the association of self-reported consumption of cooked meat, fish, sea food, and eggs with PCa risk among Nigerians in a case-control design.

## Methods

**Figure 1:**
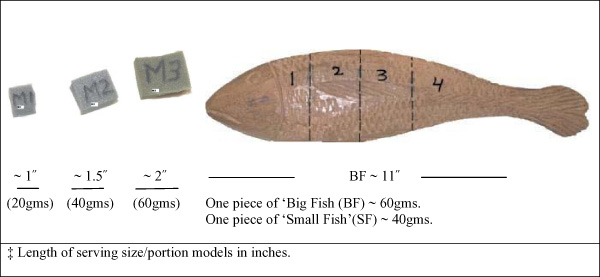
Models of serving sizes^‡ ^and portions of meat (M1, M2, M3) and fish (1, 2, 3, 4) utilized in interviewing participants in the Nigerian study population.

Men 40 years and older recruited by door-to-door invitation from two rural and two urban communities of Edo and Delta states of Southern Nigeria, were screened for PCa by PSA blood test and DRE examination. Also men attending the surgery and urology clinics of the University of Benin Teaching Hospital with prostate related complaints were also recruited. Trained interviewers obtained informed consent and completed the personal information questionnaire, and a food frequency inventory based on the Block FFQ modified by the addition of Nigerian foods and culturally appropriate serving portions (Figure 1) for each participnats. Participants were asked to return the next day without taking breakfast, and at the second visit 30 ml fasting venous blood was collected into three tubes, a urology symptom history and digital rectal examination was conducted by a surgeon/urologist, and their physical body fat parameters were measured by a trained research assistant. Each participant received a cash incentive and gifts at the end of each study visit. PSA was analyzed by a commercial laboratory in the US. The PCa cases were histologically confirmed, and the controls were men with normal sized prostates with a PSA <4 ngs/ml.

Annual frequencies for red, white and organ meat, fish, and sea food intake were computed by adding annual frequency for each food item in that group. Food-group annual frequency <18 was labeled 'Rarely', 18–181 'Sometimes', and ≥ 182 'Frequently'. Annual quantity consumed was computed by multiplying annual frequency by unit portion size as described in Table 1. Demographic and other characteristics of PCa cases and controls were compared using Chi-square test, and odds ratio and 95% confidence interval [OR(95%CI)] of PCa risk for food items estimated by unconditional logistic regression controlling for age and educational status. 591(87.8%) of 673 consented men completed the FFQ, and dietary risk assessment was based on the 374 entries in the current data base.

**Table 1:** Computing annual intake of food items by multiplying average self-reported annual frequency of food intake by unit portion size

Annual Intake Pattern	Never	Rarely	Occasionally	Sometimes	Frequently	Every Day (Daily)	Many Times (A Lot)
Reference	0/Year	≤6/Year	1–2/Month	1–2/Week	3–4/Week	5–7/Week	≥ 2/Day
Interval Boundary	0						365 × 2
Mid-Interval Value	0	3	18	78	182	312	730
Frequency (3 Groups)	Rarely		Sometimes		Frequently		
Annual Intake	Mid-Interval Frequency × Unit Portion Size						
Annual Intake (4 Groups)	Transform to Quartiles						

## Results

A total of 591 participants participated in this study, 334(56.5%) recruited from the community and 257(43.5%) from hospital clinics. There were 140 (23.7%) PCa cases, 78 (13.2%) with elevated PSA, and 373(63.1%) controls with mean ages 70.10 ± 10.6, 67.0 ± 10.9, and 56.09 ± 12.1 respectively, *P *< 0.0001. 127(20.1%) of the controls were recruited from the clinics and 293(79.9%) from the community. The characteristics of the 324 with their FFQ information in the database are presented in Table 2. Forty-five percent (45.9%) ate fresh fish, 43.3% beef, 16.9% eggs, and 7.2% chicken frequently, at least 3–4 times per week. The rate of frequent consumption of food groups was 227(71.6%) for fish/sea food, 198(62.3%) for red meat, 48(15.0%) for white meat, and 37(11.6%) for organ meat. Frequency pattern for meat, fish, and eggs were statistically different by education status, age, and urban/rural residency, but not by income group. The usual serving portion for fish was <40 gms for 72.4% of the participants, ≤ 60 gms of beef (51.0%), one piece of chicken (89.5%), and ≤ 2 eggs (84.6%). Pattern of intake of fish/seafood, white meat, organ meat and eggs were similar for cases and controls. Cases ate red meat (48.2% vs. 64.9%, p < 0.07), and shrimp (19.6% vs. 24.6%, p < 0.01) less frequently than controls (Table 3). PCa risk trend comparing 4^th ^to 1^st ^quartile annual intake was significant for red and organ meat, p < 0.04, with OR(95% CI) 1.74(0.59–5.17), 0.94(0.34–2.64), 1.16(0.50–2.68), and 1.18(0.50–2.81) for red meat, organ meat, fish and egg respectively (Table 4). In the study sample 2.2%, 7.1%, 8.1%, and 12.6% reported that they did not eat red meat, chicken, fish, and eggs respectively in the previous year.

**Table 2:** Demographic and other characteristics of prostate cancer cases and controls in the Nigerian study population

Characteristic	Cases n = 56	Controls n = 268	*p*-value
Residency			<0.04
Rural	25(44.6)	161(60.1)	
Urban	31(55.4)	107(39.9)	
			
Recruitment site			<0.001
Community	12(21.4)	251(93.7)	
Hospital Clinics	44(78.6)	17(6.3)	
			
Age (years)			<0.001
<54	3(5.4)	137(51.1)	
55–74	32(57.1)	112(41.8)	
≥ 75	21(37.5)	19(7.1)	
			
Education			<0.03
None	19(33.9)	54(20.1)	
<Secondary	23(41.1)	122(45.5)	
Secondary	1(1.8)	41(15.3)	
Post-Secondary	6(10.7)	25(9.3)	
College	7(12.5)	26(9.7)	
			
Annual Income (Naira)^§^			ns
<N45,000	47(90.4)	175(77.1)	
N45,000–N85,000	3(5.8)	20(8.8)	
≥ N85,000	2(3.8)	32(14.1)	
			
History of BPH			<0.001
Self-Report	22(39.3)	11(4.1)	
			
Obesity status			
BMI ≥ 30 (kg/m^2^)	7(14.0)	16(6.2)	ns
BMI ≥ 35 (kg/m^2^)	2(4.0)	32(14.1)	ns
			
Anthropometry (Mean)			
WHR	0.97 ± 0.09	0.92 ± 0.07	<0.001
BMI (kg/m^2^)	23.9 ± 5.15	23.4 ± 3.84	ns
Height (cm)	165.1 ± 9.37	166.8 ± 7.60	ns
Skin fold thickness^‡^(mm)	8.9 ± 4.19	8.9 ± 4.09	ns

§ Nigerian currency

‡ Average skin fold ((biceps + triceps + sub-scapular)/3)

**Table 3:** Rate of frequent^‡ ^intake of meat, fish and eggs among prostate cancer cases and controls in the Nigerian study population

	Frequency (%)		
Food item	Cases	Controls	*p*-value
Chicken	4(7.1)	19(7.1)	0.67
Turkey	5(8.9)	15(5.6)	0.13
Pork	1(1.9)	18(6.7)	0.06
All white meat	7(12.7)	41(15.3)	0.67
			
Beef	17(30.4)	123(45.9)	0.08
Goat	1(1.8)	16(6.0)	0.18
Game	4(7.1)	36(13.4)	0.13
Skin	9(16.1)	52(19.6)	0.28
All red meat	27(48.2)	172(64.9)	0.07
			
Kidney/Liver	1(1.8)	22(8.9)	0.12
Gizzard	1(1.8)	5(1.9)	0.52
Tripe	0(0.0)	9(3.4)	0.30
Organ meat	4(7.1)	33(12.3)	0.09
			
Fresh fish	24(42.9)	122(45.5)	0.90
Dry fish	17(30.4)	92(34.3)	0.71
All fish	30(53.6)	168(62.7)	0.45
			
Shrimp	11(19.6)	66(24.6)	0.01
Crab	2(3.6)	16(6.0)	0.47
Snail	1(1.8)	14(5.2)	0.29
Fish & sea food	33(58.9)	195(72.8)	0.13
			
Egg	11(19.6)	43(16.0)	0.21

‡ Self-reported food frequency ≥ 3 times per week

**Table 4:** Odds ratios and 95% confidence interval (CI) for prostate cancer risk comparing lowest to highest quartiles of dietary intake of meat, fish, seafood and eggs in the Nigerian study population

	Annual Intake Quartiles Odds Ratio(95%CI)				
Food item	Q1	Q2	Q3	Q4	*p *for trend
Red meat	1.00	0.46(0.20–1.09)	0.60(0.25–1.45)	1.74(0.59–5.17)	0.04
White meat	1.00	0.71(0.31–1.62)	0.71(0.31–1.62)	1.13(0.45–2.86)	0.67
Organ meat	1.00	0.34(0.13–0.83)	0.50(0.19–1.31)	0.94(0.34–2.64)	0.04
All meat	1.00	0.69(0.30–1.57)	1.21(0.49–2.98)	2.95(0.96–9.10)	0.06
Fish	1.00	1.14(0.49–2.66)	2.41(0.91–6.42)	1.16(0.50–2.68)	0.86
All sea food	1.00	0.94(0.39–2.24)	0.75(0.32–1.76)	1.33(0.51–3.48)	0.67
Eggs	1.00	0.94(0.39–2.31)	1.34(0.52–3.45)	1.18(0.50–2.81)	0.86

## Discussion

Red meat is one of the main content of western diet proposed as a modifiable risk factor for PCa [14]. The increase in PCa incidence in Japan in the 1980s [2], and sub-Saharan Africa more recently, has been attributed to transition from the traditional low-animal fat diet to a 'westernized' diet high in animal fat, leading to modification of the natural history of PCa [12,13,15,16]. Unlike other studies that reported strong associations with red meat [14] and organ meat intake [17], our study demonstrated only a modest increased risk trend across quartiles of red and organ meat intake, but the OR for risk was not statistically significant. The fact that meat is usually boiled in this population may explain the attenuated effect of red meat since carcinogens are produced by grilling and frying [6,18]. Our findings are consistent with other reports that did not demonstrate PCa risk association with total meat, white meat [19], and egg intake [20].

Fish is the main source of protein for shoreline Africans such as Nigerians [21,22], and is more popular than meat in this population. The three commonly eaten fish are the saltwater croaker and mackerel, and the fresh water catfish, usually dried, broiled, and sometimes fried. Our data did not support the negative association between fish intake and PCa risk as reported in the study of Native Alaskan Eskimos who eat large quantities of fish [23]. Similarly a cohort study in Japan did not find PCa risk association with fish intake among men 40–69 years [24]. Japanese traditional diet, high in soybean and fish, is associated with low PCa risk [25], underscoring the importance of an entire dietary style over individual food items.

Recall error associated with the FFQ may be limited in this study given the homogeneous nature of Nigerian diet, and exposure misclassification was reduced by the use of life-size food portion models. We did not transform portion size units to actual weight, and this might attenuate statistical association if between-person differences in portion size contribute to between-person variability in amount consumed. We also did not collect information about the type of fish eaten, which together with method of preparation might be very important in cancer etiology. Despite these limitations we have no reason to disagree with the hypothesis postulated by other authors that high intake of red meat contributes to PCa risk. We however had no evidence to support the hypothesis that high intake of fish reduces PCa risk. In the absence of nutrient composition tables of Nigerian foods, we have reported preliminary results of PCa risk associations of selected food items acknowledging the limitations of FFQ in cancer risk assessment.

## Conclusion

This study examined the association of self-reported consumption of cooked meat, fish, sea food, and eggs with PCa risk among Nigerians. Fish is more popular in the Nigerian population, followed by red meat, while chicken and eggs are not popular food items. The overall serving portions reported by participants are very modest. Our data did not demonstrate statistical association between frequent consumption of fish, seafood, and eggs, red, white and organ meat with PCa risk. However, consistent with previous reports, there was a modest significant increased risk trend for men in the upper quartile of quantity of red meat consumed. In contrast to other reports we did not observe any risk reduction with the quantity of fish consumed. These preliminary findings need to be confirmed in a large study sample, and future research should investigate the impact of westernized dietary transition on the development of PCa in a designated low-incidence region such as Nigeria.

## Competing interests

The authors declare that they have no competing interests.

## Authors' contributions

FAU conceived, developed, designed, and coordinated the study, trained research staff, performed statistical analysis, and developed the manuscript. KT participated in statistical data analysis and drafted the manuscript, EE coordinated data collection in Nigeria, ML was responsible for data entry, TO, PA, and UO examined study participants (cases and controls) and provided access to their patients, DB helped to interpret the data and to draft the manuscript. All authors read and approved the final manuscript.
